# Accuracy of automated segmentation and volumetry of acute intracerebral hemorrhage following minimally invasive surgery using a patch-based convolutional neural network in a small dataset

**DOI:** 10.1007/s00234-024-03311-4

**Published:** 2024-02-17

**Authors:** Samer Elsheikh, Ahmed Elbaz, Alexander Rau, Theo Demerath, Christian Fung, Elias Kellner, Horst Urbach, Marco Reisert

**Affiliations:** 1 Department of Neuroradiology, Faculty of Medicine, Medical Center - University of Freiburg, Breisacherstr. 64, 79106 Freiburg, Germany; 2 Department of Diagnostic and Interventional Radiology, Faculty of Medicine, Medical Center - University of Freiburg, Freiburg, Germany; 3 Department of Neurosurgery, Faculty of Medicine, Medical Center - University of Freiburg, Freiburg, Germany; 4 Medical Physics, Department of Diagnostic and Interventional Radiology, Medical Center, Faculty of Medicine, University of Freiburg, Freiburg, Germany; 5 Department of Stereotactic and Functional Neurosurgery, Faculty of Medicine, Medical Center - University of Freiburg, Freiburg, Germany

**Keywords:** Intracerebral hemorrhage, Minimally invasive surgery, Machine learning, Automated volumetry, Convolutional neural network

## Abstract

**Purpose:**

In cases of acute intracerebral hemorrhage (ICH) volume estimation is of prognostic and therapeutic value following minimally invasive surgery (MIS). The ABC/2 method is widely used, but suffers from inaccuracies and is time consuming. Supervised machine learning using convolutional neural networks (CNN), trained on large datasets, is suitable for segmentation tasks in medical imaging. Our objective was to develop a CNN based machine learning model for the segmentation of ICH and of the drain and volumetry of ICH following MIS of acute supratentorial ICH on a relatively small dataset.

**Methods:**

Ninety two scans were assigned to training (n = 29 scans), validation (n = 4 scans) and testing (n = 59 scans) datasets. The mean age (SD) was 70 (± 13.56) years. Male patients were 36. A hierarchical, patch-based CNN for segmentation of ICH and drain was trained. Volume of ICH was calculated from the segmentation mask.

**Results:**

The best performing model achieved a Dice similarity coefficient of 0.86 and 0.91 for the ICH and drain respectively. Automated ICH volumetry yielded high agreement with ground truth (Intraclass correlation coefficient = 0.94 [95% CI: 0.91, 0.97]). Average difference in the ICH volume was 1.33 mL.

**Conclusion:**

Using a relatively small dataset, originating from different CT-scanners and with heterogeneous voxel dimensions, we applied a patch-based CNN framework and successfully developed a machine learning model, which accurately segments the intracerebral hemorrhage (ICH) and the drains. This provides automated and accurate volumetry of the bleeding in acute ICH treated with minimally invasive surgery.

## Introduction

The global burden of intracerebral hemorrhage (ICH) is estimated at about 5 million events annually and has a high morbidity and mortality [1]. Minimally invasive surgery (MIS) for hematoma evacuation aims to reduce ICH volume and perihematomal edema. Current guidelines recommend MIS in patients with supratentorial ICH > 20–30 ml volume and Glasgow coma scale of 5–12 [2]. The MISTIE III trial is the largest study on MIS to date and identifies a reduction of the clot size to 15 ml or less and a correct drain position within the ICH as the aim for the procedure [3]. Therefore, estimation of ICH volume is of therapeutic relevance.

In general, the ABC/2 method is widely established to measure volumes in CT scans. It was validated in different clinical settings e.g. neoplasia [4–6]. Nevertheless, other investigations identified significant deviation from manual planimetric methods, especially in irregularly shaped objects [7, 8]. Planimetric volumetry of ICH is a time intensive task averaging roughly 3.4 min per patient [9].

Advancements in supervised machine learning using convolutional neural networks (CNN) for automated segmentation have demonstrated high accuracy in detecting, classifying, and segmenting ICH. However, these studies required large datasets of 300, 600 and 1732 scans respectively [9–11]. In contrast, hierarchical, patch-based CNN architectures, trained on smaller datasets, enable segmentation in large 3D images, exhibiting encouraging results in complex segmentation tasks [12–14].

In this study, our objective was to develop a machine learning algorithm for the segmentation of ICH and of the drain and volumetry of ICH subsequent to minimally invasive surgery of acute supratentorial ICH.

## Materials and methods

Approval of the institutional review board was obtained and the requirement for informed consent was waived. We selected patients suffering from supratentorial ICH that were treated with MIS from a retrospective database. Inclusion criteria were age ≥ 18 years and available CT-imaging. No exclusions were made based on scanner model, settings, voxel size or presence of artefacts.

Ground truth (GT) annotation and development of the CNN were carried out using a local instance of the Nora imaging platform (https://www.nora-imaging.com). Image calculations were done using MATLAB (MATLAB R2021a, The MathWorks). Statistical evaluation of the results and plotting were done using R software version 4.2.0 [15].

### Imaging datasets

Thirty nine scans from 29 patients examined between years 2011 and 2018 were randomly selected from our database. To avoid data leakage, we partitioned our data on the patient level, thus ensuring that repeat examinations of all patients were assigned to the same group. We randomly divided the data into training (n = 21 patients / 29 scans), validation (n = 3 patients / 4 scans) and testing (n = 5 patients / 6 scans). To avoid the effect of random patient selection on the results, we added not yet included consecutive patients examined between 2010—2012 to the testing dataset for a total of 59 scans belonging to 44 patients. The mean age (SD) was 70 (± 13.56) years and there were 36 male patients (52.9%).

### Ground truth

Non-overlapping segmentation masks of the ICH and the intracranial part of the drain were manually delineated by a neuroradiologist, with three years of experience (A.E.). Overlapping voxels were subsequently identified and classified to their corresponding mask by applying a threshold operation with voxels ≥ 100 HU assigned to the drain mask.

### CNN segmentation of ICH and drain

No preprocessing was applied to the CT data. The development of the CNN model relied on the Patchwork CNN Toolbox [12]. Here, the input for the CNN was the CT image in HU units. Instead of normalizing/cropping the image, an initial channel splitting layer was used. This channel splitting layer separates the input value range into 11 feature channels that are sensitive to a particular HU range. This method was inspired by the windowing approach that a radiologist uses when reading images by dividing the entire HU area into detachable image parts, e.g., CT windows for soft tissue or bone. The ranges are initialized with the following centers [-1000, -500, -100, -50, -25, 0, 25, 50, 100, 500, 1000], and further refined during training. Three hierarchical scales (patches) were used. The finest scale was reformatted to 1-mm isotropic voxels.

To determine the best model parameters we initially tested six different combinations on 10^6^ image patches, experimenting with two different versions of three model parameters.

1. Feature dimensions in each scale: [8, 16, 16, 32, 64] or [8, 16, 16, 32, 64, 64]
2. Loss function: categorical or binary cross-entropy.
3. Augmentation at each level of the network: rotation angle 0.2, right-left flipping and zooming 10–20% or rotation angle 0.4, flipping in all dimensions, zooming 10–20% and random uniform scaling of the voxel values in each scale.

### Performance measures

We employed the Deepmind library (https://github.com/deepmind/surface-distance) to measure overlap and spatial distance metrics.

1. Dice similarity coefficient (DSC) which measures the overlap of two sets of points
2. Surface DSC, which measures the overlap of the surfaces of two sets of points at a specific tolerance (1 mm). The surface DSC is better suited than DSC for assessing performance in 3D segmentation tasks [16].
3. Surface overlap measures the average overlap at a specific tolerance (1 mm) returning two values. The average overlap from the GT surface to the predicted surface and vice versa.
4. Hausdorff distance measures the distance between two sets of points. To alleviate its sensitivity to outliers, both the Hausdorff100 and Hausdorff95 (top 95% of the distances are taken into account) were evaluated.
5. Average surface distance, which measures the distances between the surfaces of two sets of points at a specific tolerance (1 mm) and thus returning two values. The average distance from the GT surface to the predicted surface and vice versa [16, 17].

The top-performing model on the validation dataset was trained using 1.2 × 10^7^ patches. The model output is a 4D NIfTI object with two 3D 1-mm isotropic NIfTI volumes, indicating the probability of each voxel belonging to ICH/drain or to the background. Binary masks were produced using a threshold to optimize performance measures of the CNN. The volume of ICH was calculated by summing the 1-mm isotropic voxels of the ICH mask.

### Comparison with no-new-U-Net (nnU-Net)

Isensee et al. [18] published a self-adapting semantic segmentation method that was tested on a wide variety of medical imaging datasets with good results, as well as achieving top placements in multiple segmentation challenges. Using the same data partitions, we trained and tested an nnU-Net model using our datasets.

### Statistical tests

To assess the agreement between predicted and GT ICH volume we calculated the intraclass correlation coefficient (ICC) [19]. We also generated concordance plots and Bland–Altman plots [20] to visualize the agreement between the two measurements.

## Results

### Imaging characteristics

Images were acquired from a single center on three different scanners. Range of voxel sizes was 0.38–0.52 × 0.38–0.52 × 0.7–5 mm^3^. Soft kernel reconstructions were available in all patients.

### CNN segmentation patchwork results

**Fig. 1:**
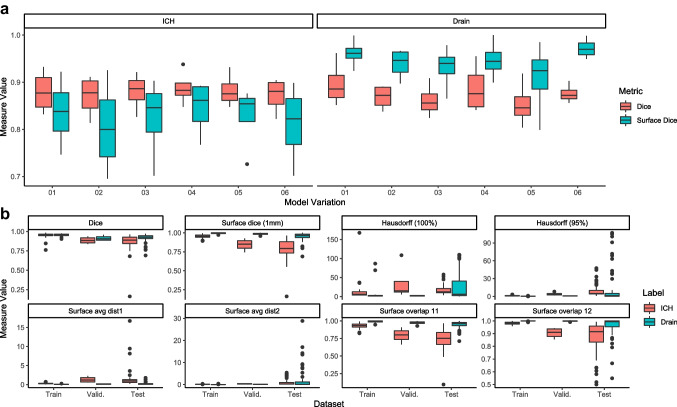
**a**: Dice and surface dice coefficients in all model variations in validation dataset. **b**: Similarity and overlap metrics of the final model variation in all datasets

We tested six different model variations to approximate optimal parameter settings. Here, we selected the best performing model based on the DSC and the surface DSC. Models 1 and 4 showed the best performance (Table 1, Fig. 1). These model parameters were those trained using less complex parameter variants, suggesting overfitting with more complex model architectures. Model 1 employed a categorical cross-entropy, while model 4 utilized a binary cross-entropy loss function. A minimal advantage was observed with model 4 compared to model 1. We thus selected the parameters of model 4 and trained our final model using 1.2 × 10^7^ patches, which resulted in sufficient overlap metrics (Table 2, Fig. 1).

**Table 1:** DSC and Surface DSC at tolerance of 1 mm of ICH and drain of all model variations in training and validation datasets

Model	Metric (Tolerance)	ICH Train	ICH Validation	Drain Train	Drain Validation
01	Dice	0.920	0.880	0.920	0.896
01	Surface dice (1 mm)	0.890	0.836	0.972	0.962
02	Dice	0.890	0.870	0.879	0.868
02	Surface dice (1 mm)	0.817	0.805	0.928	0.939
03	Dice	0.891	0.880	0.859	0.861
03	Surface dice (1 mm)	0.823	0.824	0.908	0.931
04	Dice	0.921	0.888	0.919	0.887
04	Surface dice (1 mm)	0.889	0.846	0.969	0.947
05	Dice	0.898	0.883	0.846	0.854
05	Surface dice (1 mm)	0.827	0.828	0.880	0.908
06	Dice	0.889	0.872	0.878	0.876
06	Surface dice (1 mm)	0.814	0.811	0.932	0.972

**Table 2:** Performance measures of the final model in ICH and drain across all datasets

Metric (Tolerance)	ICH Train	ICH Validation	ICH Test	Drain Train	Drain Validation	Drain Test
Dice	0.95	0.89	0.86	0.95	0.91	0.91
Hausdorff (100%)	14.85	36.19	16.66	7.31	2.01	26.77
Hausdorff (95%)	1.23	4.07	9.04	0.44	0.79	14.44
Surface avg dist 1	0.29	1.29	1.52	0.06	0.13	0.24
Surface avg dist 2	0.12	0.33	0.88	0.04	0.05	2.19
Surface dice (1 mm)	0.95	0.84	0.79	0.99	0.98	0.95
Surface overlap 1 (1 mm)	0.93	0.79	0.74	0.99	0.97	0.94
Surface overlap 2 (1 mm)	0.98	0.90	0.87	1.00	1.00	0.96

**Fig. 2:**
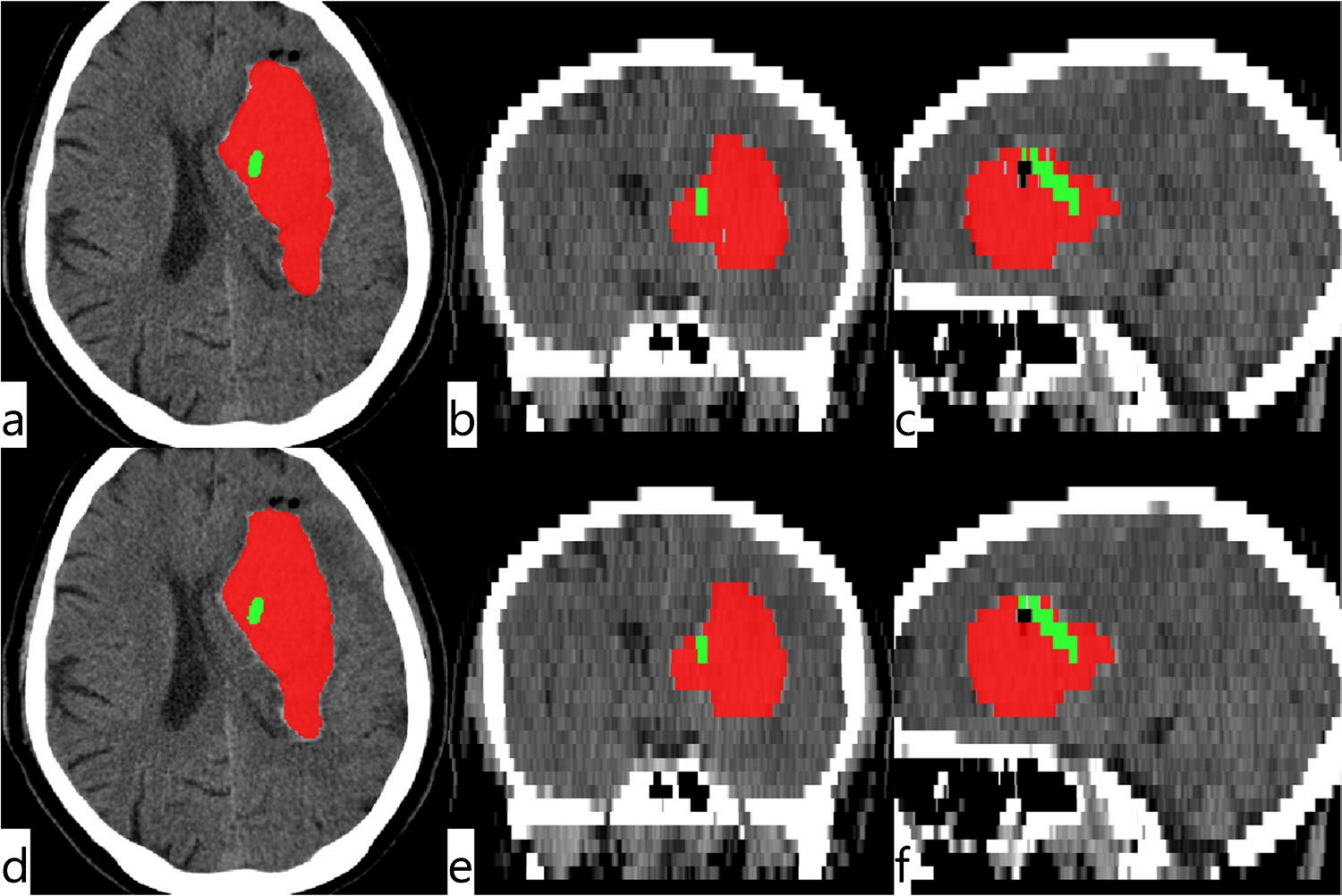
CT images of a test patient. a & d axial, b & e coronal and c & f sagittal reformats of the CT scan with GT (top row) and predicted masks (bottom row) of ICH (red) and the drain (green)

Three dimensional binary masks for the ICH and the drain were produced from the model output using a probability threshold of 0.5. The resulting NIfTI objects are in the same reference space as the CT images, facilitating superimposition, visualization, and export to PACS systems (Fig. 2).

### Segmentation result using nnU-Net

We trained an nnU-Net (https://github.com/MIC-DKFZ/nnUNet) model using default settings. Table 3 shows the results in all datasets.

**Table 3:** Performance measures of nnU-Net in ICH and drain across all datasets

Metric (Tolerance)	ICH Train	ICH Validation	ICH Test	Drain Train	Drain Validation	Drain Test
Dice	0.95	0.95	0.87	0.97	0.96	0.94
Hausdorff (100%)	14.96	33.39	16.94	3.70	0.71	4.97
Hausdorff (95%)	1.44	1.24	6.81	2.30	0.21	3.03
Surface avg dist 1	0.32	0.74	1.23	0.03	0.03	0.08
Surface avg dist 2	0.16	0.11	0.58	0.47	0.03	1.00
Surface dice (1 mm)	0.94	0.96	0.81	0.99	1.00	0.98
Surface overlap 1 (1 mm)	0.92	0.93	0.75	1.00	1.00	0.99
Surface overlap 2 (1 mm)	0.97	0.98	0.90	0.99	1.00	0.98

### ICH volumetry patchwork results

**Fig. 3:**
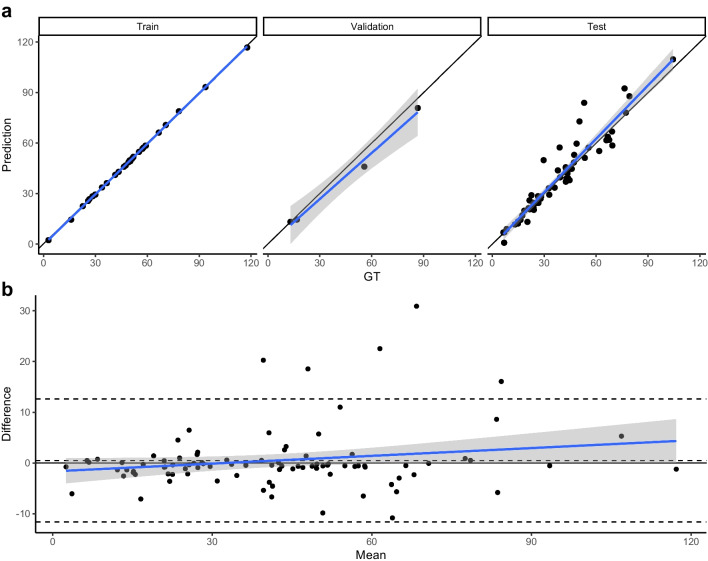
**a**: Concordance plot of GT and predicted ICH volumes in all patients in the CNN dataset. Regression line (blue) and 95% confidence interval of predicted values. **b**: Bland–Altman plot of GT and predicted ICH volumes in all patients. Regression line (blue) and 95% confidence interval of differences

The mean (± SD) of GT ICH volumes in the training, validation and testing datasets were 49 (± 23.1), 42.9 (± 34.9), and 37.8 (± 21.1) mL respectively. The mean (± SD) predicted ICH volumes were 48.5 (± 23.1), 38.5 (± 31.9) and 39.1 (± 23.5) mL respectively. ICC was calculated, which showed an excellent agreement of 0.94 (95% CI: 0.91, 0.97) in the test dataset. Figure 3 depicts ICH volume concordance plots and Bland–Altman plots, both showing excellent agreement between predicted and GT ICH volumes across all values. In the test dataset, our model prediction overestimated the ICH volume on average by 1.33 mL.

### ICH volumetry using nnU-net

Automated volumetry using the predictions of the nnU-Net model yielded an ICC of 0.96 (95% CI: 0.94, 0.98) between the GT volumes and the predicted volumes in the continuous testing data set.

## Discussion

We developed a CNN machine learning model to segment ICH and drains in cases treated with minimally invasive surgery. Our model accurately segmented the ICH and drain with DSC scores of 0.86 and 0.91 respectively. Additionally, automated ICH volumetry yielded high agreement with ground truth (ICC = 0.94 [95% CI: 0.91, 0.97]), overestimating the ICH volume by 1.33 mL. We developed our model with relatively small training and validation datasets (n = 33) of heterogeneous data, originating from various scanners, a wide range of voxel sizes, and anisotropy, which enhances the model’s generalizability. Moreover, we did not employ image preprocessing, minimizing processing power demands, and making the model independent of preprocessing algorithm results such as skull stripping or cropping.

As ICH demonstrates excellent contrast in CT, it has been utilized for automated diagnosis with different levels of success. Most of the previous research focused on detecting ICH and reported accuracy measures reaching 0.98 for area under the receiver operating characteristic curve [21]. In our study, all cases suffered an ICH and all were successfully detected. However, this was not the purpose of our study and we acknowledge a conceivable selection bias in our cohort. As we only included patients selected for MIS, the ICH volume in our cohort may have been skewed towards larger volume (41.5 mL). The average ICH volume in our cohort, while comparable to that reported in the MISTIE III Trial (47.4 mL) [10], was higher compared to studies focusing on ICH segmentation. For instance, Ironside et.al. reported an average volume of 25.7 mL [9].

In our study, we applied a patch based CNN toolbox [12], which allows model development by creating training patches in the magnitude of millions from a small number of scans (n = 33 in our study). Testing on a larger test dataset (n = 59) resulted in a sufficient model performance (DSC = 0.86). The results are comparable with other published works, which reached a DSC of 0.92 training on several hundreds of scans [11, 22–27]. Applying the no-new-Unet segmentation toolbox [18] to our dataset yielded a good result (DSC = 0.87) that is comparable to our model. We assume that the sufficient results in spite of using a small dataset, may be attributed to the excellent image contrast in this segmentation task.

Volume reduction of ICH on follow up examinations is an aim of the treatment according to the MISTI III study and has been linked to 12-month mortality [3, 28]. Accurate automated volumetry could address the shortcomings of the ABC/2 approach. However, to accomplish this, an accurate segmentation of the hemorrhage is required. We achieved a very high correlation between predicted and GT ICH volume (ICC = 0.94 [95% CI: 0.91, 0.97]), which lies within the range of other automated algorithms, where a comparable correlation coefficient reaching 0.98 was achieved [11, 23, 24, 26, 27].

Furthermore, we achieved accurate segmentation of the drain (DSI = 0.91). A literature search yielded no other articles that attempt to segment drains following MIS. Precise segmentation of the ICH and drain might potentially simplify the evaluation of the drain position following MIS, which is crucial for treatment success.

## Conclusions

Using a relatively small dataset, originating from different CT-scanners and with heterogeneous voxel dimensions, we applied a patch-based CNN framework and successfully developed a machine learning model, which accurately segments the intracerebral hemorrhage (ICH) and the drains. This provides automated and accurate volumetry of the bleeding in acute ICH treated with minimally invasive surgery.

## Data Availability

Data are available from the authors upon reasonable request and approval of the local ethics committee.

## Code availability

A standalone version of the trained model and a script for prediction are available in a Github repository (Github: https://github.com/s-elsheikh/segment_ich).
